# Bullous Cutaneous Eruption due to Extravasation of Acyclovir in an Adolescent with Acute Lymphoblastic Leukemia

**DOI:** 10.5505/tjh.2012.93685

**Published:** 2012-03-05

**Authors:** Abdurrahman Sarıca

**Affiliations:** 1 Dr Abdurrahman Yurtaslan Oncology Education and Research Hospital, Ankara, Turkey

A 14-year-old-girl undergoing treatment with the BFMTRALL 2000 protocol (MRG ) because of early pre-B-cellacute lymphoblastic leukemia (ALL ) developed chickenpox on the 33rd d of remission induction. As such, chemotherapywas withdrawn and the patient was quarantined.Acyclovir (1500 mg·m–2·d–1 in three divided doses)was then administered in 100 mL of 0.9% sodium chloride,as 1-h intravenous infusions. On the 9th d of acyclovirtherapy 10 min after infusion of the 27th dose startedthe patient complained of the sensation of minor pain andburning in the region of Intracath catheter insertion, anddeveloped slight erythema with irregular boundaries inthe same region.

The infusion line was checked for patency by gentlywithdrawing blood before, which showed that the linewas patent, and then infusion was continued, but at aslower rate. The line’s patency was checked frequently andremained patent. The patient no longer experienced thesensation of pain and burning, and the erythema improvedslightly; however, at the end of the infusion (the 65th min)a solitary, bullous painless eruption 1 x 1 cm in diameterwas observed on the tract of the vein, 10 cm distal of theIntracath insertion (Figures 1,2,3,4). Without any medicalintervention the lesion subsided in 8 h and disappearedcompletely in 24 h, leaving behind a residual scar lesion.

The known adverse dermatological effects of acyclovir,including erythema, inflammation, and phlebitis at the siteof intravenous infusion, occur in ≤16% of patients, presumablydue to the alkaline nature of the solution (reconstitutedacyclovir has a pH of 10-11) [1]. It is a knownirritant to venous and soft tissue if extravasated [2].Wethink that both the erythema and bullous eruption inthe presented case were signs of subcutaneous acyclovirextravasation, despite the fact that we frequently checkedthe line and were confident of its patency.

Frequent venipuncture of the same veins and use ofchemotherapeutic agents in oncology patients may rendertheir veins fragile and susceptible to the irritant effects ofdrugs. As such, sensation of pain and burning in oncologypatients should be considered a reliable sign of extravasationeven when good blood return is observed. Nonetheless,cutaneous vesicular eruption following intravenousacyclovir administration is rare [1]. It was reported thatcutaneous vesicular eruptions developed not only at thesite of injection, but far from it [3] and proximal to it [1].Moreover, bullous eruptions were also reported followingtopical and oral acyclovir administration [1]; therefore,rather than extravasation of acyclovir solution, an immunoallergicpattern in the presence of histological leukocytoclasticvasculitis is an etiological consideration [3].

The presented case shows that the irritant effects ofacyclovir should always be a consideration, especially inoncology patients, and that the development of new vesiculareruptions during acyclovir therapy should not alwaysbe considered progression of herpes infection.

**Conflict of Interest Statement**

The authors of this paper have no conflicts of interest,including specific financial interests, relationships, and/or affiliations relevant to the subject matter or materialsincluded.

**Acknowledgement**

I thank Lale Olcay, MD for kindly revising the manuscript.

## Figures and Tables

**Figure 1:**
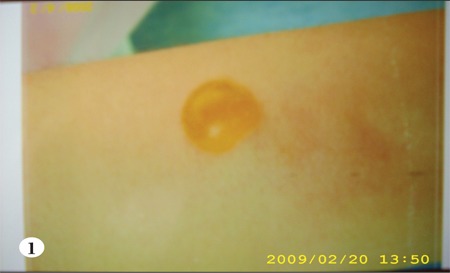
Cutaneous vesicular eruption on the forearm ofthe patient which developed after acyclovir injection.

**Figure 2:**
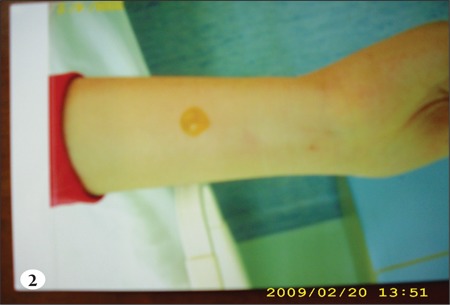
Cutaneous vesicular eruption on the forearm ofthe patient which developed after acyclovir injection.

**Figure 3:**
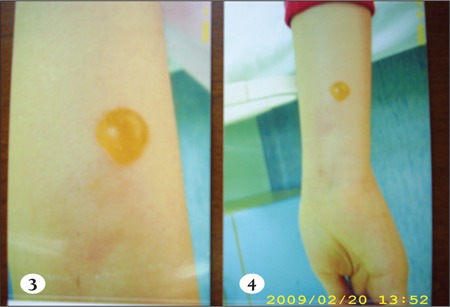
Cutaneous vesicular eruption on the forearm ofthe patient which developed after acyclovir injection.
